# Identification of Genome-Wide Copy Number Variations among Diverse Pig Breeds Using SNP Genotyping Arrays

**DOI:** 10.1371/journal.pone.0068683

**Published:** 2013-07-23

**Authors:** Jiying Wang, Haifei Wang, Jicai Jiang, Huimin Kang, Xiaotian Feng, Qin Zhang, Jian-Feng Liu

**Affiliations:** 1 Key Laboratory of Animal Genetics, Breeding and Reproduction, Ministry of Agriculture, National Engineering Laboratory for Animal Breeding, College of Animal Science and Technology, China Agricultural University, Beijing, China; 2 Shandong Provincial Key Laboratory of Animal Disease Control and Breeding, Institute of Animal Science and Veterinary Medicine, Shandong Academy of Agricultural Sciences, Jinan, China; Wageningen UR Livestock Research, Netherlands

## Abstract

Copy number variations (CNVs) are important forms of genetic variation complementary to SNPs, and can be considered as promising markers for some phenotypic and economically important traits or diseases susceptibility in domestic animals. In the present study, we performed a genome-wide CNV identification in 14 individuals selected from diverse populations, including six types of Chinese indigenous breeds, one Asian wild boar population, as well as three modern commercial foreign breeds. We identified 63 CNVRs in total, which covered 9.98 Mb of polymorphic sequence and corresponded to 0.36% of the genome sequence. The length of these CNVRs ranged from 3.20 to 827.21 kb, with an average of 158.37 kb and a median of 97.85 kb. Functional annotation revealed these identified CNVR have important molecular function, and may play an important role in exploring the genetic basis of phenotypic variability and disease susceptibility among pigs. Additionally, to confirm these potential CNVRs, we performed qPCR for 12 randomly selected CNVRs and 8 of them (66.67%) were confirmed successfully. CNVs detected in diverse populations herein are essential complementary to the CNV map in the pig genome, which provide an important resource for studies of genomic variation and the association between various economically important traits and CNVs.

## Introduction

Copy number variations (CNVs) are gains and losses of genomic sequence greater than 50 bp between two individuals of a species [1], [2]. The milestone work by Iafrate et al. and Sebat et al. 2004 [3], [4] firstly revealed CNVs distribute ubiquitously in the human genome. Since then, thousands of novel CNVs were detected in the human genome [5], [6], [7]. Compared with the most frequent SNP marker, CNVs cover wider genomic regions in terms of total bases involved and have potentially larger effects by changing gene structure and dosage, alternating gene regulation, exposing recessive alleles and other mechanisms [8], [9]. As an important form of genetic variation, CNVs are becoming an important source of genetic variance [10] and may account for some of the missing heritability for complex traits [11].

In domestic animals, phenotype variations caused by CNVs were also observed, for instance, the white coat phenotype in pigs caused by the duplication of the KIT gene [12], [13], the pea-comb phenotype in chickens caused by the copy number alteration in intron 1 of the SOX5 gene [13] and hair greying and melanoma in horses caused by a 4.6-kb duplication in intron 6 of STX17 [14]. Additionally, the study by Seroussi et al. [15] showed there were significant associations between the loss of this region and total merit, and between copy number of this region with the genetic evaluations for protein production, fat production and herd life. These findings demonstrate that CNVs can be considered as promising markers for some phenotypic and economically important traits or diseases in domestic animals.

Different methodologies can be applied to identify or genotype CNVs at a genome-wide scale. So far, there are three main approaches: array comparative genomic hybridization (aCGH), SNP genotyping array and high-throughput sequencing [16], [17]. Among these technologies, the SNP genotyping array has the advantage of performing both genome-wide association studies (GWAS) and CNV detection. SNP arrays can simultaneously measure both total signal intensity (Log R ratio – LRR) and allelic intensity ratios (B allele frequency – BAF) in a genomic sample, and allows both DNA copy number and copy-neutral LOH to be assessed [18]. Additionally, SNP arrays use less sample per experiment compared to aCGH, and it is a cost effective technique which allows users to increase the number of samples tested on a limited budget [19].

As one of the most economically important livestock worldwide, pig also represents one of the most important research models for various human diseases [20]. In the past few years, many efforts have been used to detect CNVs in pig genome using different technological platforms, *i.e.*, aCGH [21], [22], PorcineSNP60 BeadChip [23], [24], [25] and high-throughput sequencing [26]. Previous CNV studies in other species at genome scale suggest that CNVs comprise up to ∼12%, 4% and 4.6% of human [5], dog [27] and cattle [28] genome sequence, respectively. Compared with abundance of CNVRs detected in other species, CNVs detected in pig is far from saturation. Besides the platforms employed in CNV detection, findings from previous studies indicate that a considerable proportion of CNVs segregate among distinct breeds [2], [24], [29]. Hence, a sufficient high-resolution CNV map requires the survey of multiple breeds/populations.

Chinese indigenous breeds have larger genetic diversity and higher average heterozygosity than European breeds [30], which can help to detect fruitful breed-specific CNVs which have segregated among different populations in the course of evolution and selection. In the present study, a genome-wide CNV detection based on the PorcineSNP60 BeadChip was performed in 14 individual selected from diverse populations, including six types of Chinese indigenous breeds, one Asian wild boar population, as well as three modern commercial foreign breeds. Findings in our study have important implications for understanding the genomic variations of pig genome and provide meaningful information for association studies between CNV and economically important phenotypes of pigs in the future.

## Materials and Methods

### Animals

The whole study protocols for collection of the tissue samples of experimental individuals were reviewed and approved by the Institutional Animal Care and Use Committee (IACUC) of China Agricultural University.

According to geographic distribution and phenotypic features, the existing Chinese indigenous pig breeds have been divided into 6 distinct population types, *i.e.*, North China Type, South China Type, Central China Type, Lower Changjiang River Basin Type, Southwest Type and Plateau Type [31]. In this study, a total number of 14 individuals were chosen for SNP genotyping. These animals include one wild sow, four animals from the European breeds of Duroc (n = 2), Yorkshire (n = 1) and Landrace (n = 1) as the representatives of modern commercial breeds and nine unrelated animals selected from six Chinese indigenous breeds as the representatives of Chinese local population, including Tibetan pig (Plateau Type, n = 2), Diannan small-ear pig (South Chine Type, n = 2), Meishan pig (Lower Changjiang River Basin Type, n = 2), Min pig (North China Type, n = 1), Daweizi pig (Central China Type, n = 1), and Rongchang pig (Southwest Type, n = 1), respectively.

### SNP array genotyping and quality control

Genomic DNA of 14 individuals was extracted from the ear tissue using Qiagen DNeasy Tissue kit (Qiagen, Germany). All DNA samples were analyzed by spectrophotometry and agarose gel electrophoresis. The genotyping platform used was Infinium II Multisample assay (Illumina Inc.). SNP arrays were scanned and analyzed using iScan (Illumina Inc.) and BeadStudio (Version 3.2.2, Illumina, Inc.), respectively. The SNPs physical positions on chromosomes were derived from the swine genome sequence assembly (10.2) (http://www.ensembl.org/Sus_scrofa/Info/Index). The SNPs not mapped or mapped to multi-positions in the Sscrofa10.2 assembly were discarded. A final set of 47961 SNPs on 18 autosomes with a unique position in Sscrofa10.2 was used for further analysis.

To ensure low false positive CNVs identified, the genome-wide intensity signal must have as little noise as possible. According to our previous study [23], the high-confident CNVs can be identified with three criteria for the SNP genotyping, *i.e.*, standard deviation of normalized intensity (Log R ratio, LRR) <0.30, B allele frequency (BAF) drift <0.01 and the GC wave factor of LRR less than 0.05. In the study, all the samples were successfully genotyped with the average sample call rate higher than 99.3%, and all can satisfied the above criteria. The raw data of our SNP chip have been submitted to the Gene Expression Omnibus (http://www.ncbi.nlm.nih.gov/geo/) and released under the accession number GSE46733.

### Identification of pig CNVs

Pig CNVs were identified as previously described [23] using PennCNV software [32]. This algorithm incorporates multiple sources of information, including total signal intensity (LRR) and allelic intensity ratio (BAF) at each SNP marker, the distance between neighboring SNPs, the population frequency of B allele (PFB) of SNPs, and the pedigree information where available. Both LRR and BAF were exported from BeadStudio (Illumina Inc.) given the default clustering file for each SNP. The PFB file was calculated based on the BAF of each marker. Furthermore, PennCNV also integrates a computational approach by fitting regression models with GC content to overcome “genomic waves”. The pig gcmodel file was generated by calculating the GC content of the 1Mb genomic region surrounding each marker (500kb each side) and the genomic waves were adjusted using the *-gcmodel* option. No relationship among the sample was existed, and the pedigree/trio information was not incorporated into the analyses. To reduce the false discover rate in CNV calling, it was also required that CNV contained three or more consecutive SNPs indicating loss or gain signals. Finally, CNVs regions (CNVRs) were determined by aggregating overlapping CNVs identified across all samples according to the criteria proposed by Redon et al. [5].

Due to density limitation of SNPs on chromosome X, *i.e.* about 100 kb of averaged SNP interval, which is more than two folds of the average interval across whole genome, CNVs detected on chromosome X might had high false-positive rate and were excluded from further analyses in our study.

### Gene contents and functional annotation

Gene contents in the CNVRs identified were retrieved from the Ensembl Genes 70 Database using the BioMart (http://www.biomart.org/) data management system. Functional annotation was performed with the DAVID bioinformatics resources v6.7 (http://david.abcc.ncifcrf.gov/summary.jsp) for Gene Ontology (GO) terms [33] and Kyoto Encyclopedia of Genes and Genomes (KEGG) [34] pathway analyses. Since only a limited number of genes in the pig genome have been annotated, we firstly converted the pig Ensembl gene IDs to orthologous mouse Ensembl gene IDs by BioMart, then carried out the GO and pathway analyses. Statistical significance was assessed by using p value of a modified Fisher's exact test and Benjamini correction for multiple testing.

We also performed the overlap analyses between CNVRs identified in the study with the reported QTL regions collected in the pig QTL database (Dec 27, 2012, (http://www.animalgenome.org/cgi-bin/QTLdb/SS/index) and human disease gene orthologs in Online Mendelian Inheritance in Man annotations (OMIM, http://omim.org/).

### Quantitative real time PCR

Quantitative real time PCR (qPCR) was used to validate 12 CNVRs chosen from the total CNVRs detected in the study. The glucagon gene (GCG) is highly conserved between species and has been approved to have a single copy in animals [35]. So, one segment of it was chosen as the intern control region. Primers (Table S7 in File S1 ) were designed with the Primer3 web tool (http://frodo.wi.mit.edu/primer3/). Moreover, the UCSC In-Silico PCR tool (http://genome.ucsc.edu/cgi-bin/hgPcr?command=start) was used for in silico specificity analysis. Prior to performing the copy number assay, we generated standard curves for the primers of target and control regions to determine their PCR efficiencies. The PCR efficiencies for all primers used in the study were required to be 1.95–2.10.

All qPCR were carried out using LightCycler® 480 SYBR Green I Master on Roche LightCycler® 480 instrument following the manufacturer’s guidelines and cycling conditions. The reactions were carried out in a 96-well plate in 20 μl volume, containing 10 μl Blue-SYBR-Green mix, 1 μl forward and reverse primers (10 pM/μl) and 1μl 20 ng/μl genomic DNA. Each sample was analyzed in duplicates. The second derivative maximum algorithm included within the instrument software was used to determine cycle threshold (Ct) values for each region. The copy number for each test region was calculated using the 2^−ΔΔCt^ method [36], which compares the ΔCt (cycle threshold (Ct) of the target region minus Ct of the control region) value of samples with CNV to the ΔCt of a calibrator without CNV.

## Results

### Genome-wide detection of CNVs

**Figure 1:**
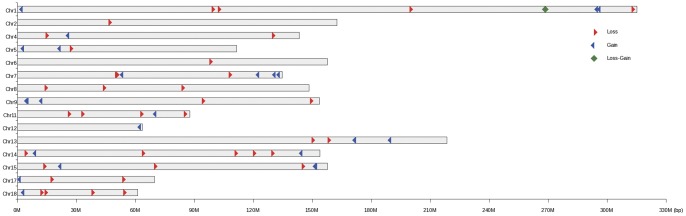
The distribution and status of detected CNVRs across the pig genome (based on the Sus scrofa 10.2 assembly).

In the study, 96 CNVs were identified on 18 autosomes using PennCNV software in 14 individual of diverse populations, including six types of Chinese indigenous breeds, one Asian wild boar population, as well as three modern commercial foreign breeds. The average number of CNVs per individual was 6.92. By aggregating overlapping CNVs, a total of 63 CNVRs (Table S1 in File S1) across genome were identified, which covered 9.98 Mb of polymorphic sequence and corresponded to 0.36% of the genome sequence. The length of these CNVRs ranged from 3.20 to 827.21 kb with a mean of 158.37 kb and a median of 97.85 kb. Among these CNVRs, there were 36 loss, 26 gain and 1 both (loss and gain within the same region) events. Figure 1 summarizes the location and characteristics of all CNVRs on autosomal chromosomes. It can be seen that these CNVRs are not uniformly distributed among different chromosomes. The length proportion of CNVRs on 18 autosomal chromosomes varies from 0–1.30%, with Chr1 harboring the greatest number of CNVRs and chr11 having the densest CNVRs.

Large difference of CNVR numbers were found among the 14 individuals (Table 1). The CNV number identified ranged from 2 (MS8) to 13 (D2), with the average of 6.92. More CNVs per individual were identified in the pigs of modern commercial breeds (10.5) than in pigs of Chinese indigenous breeds and wild population (5.5).

**Table 1:** Sample information and CNVs detected in every pig.

Types	Breed	Sample ID.	Sex	CNV number	Total Length (kb)
**Wild boar**	–	A1	Female	6	1084.98
**South Chine Type**	Diannan small-ear pig	DN1	Male	8	1732.48
		DN5	Female	6	608.40
**North China Type**	Min pig	M2	Female	5	1234.75
**Lower Changjiang River Basin Type**	Meishan pig	MS7	Female	3	1034.12
		MS8	Female	2	755.68
**Southwest Type**	Rongchang pig	R2	Male	6	874.42
**Central China Type**	Daweizi pig	W1	Female	9	1475.29
**Plateau Type**	Tibetan pig	Z2	Female	5	316.74
		Z5	Female	5	478.60
**Modern commercial breeds**	Landrace	C3	Female	10	1285.01
	Duroc	D2	Female	13	1846.16
		D4	Female	5	1536.03
	Yorkshire	Y2	Female	14	1639.64
**Mean**				6.93	1135.88

### Gene content and functional analysis

Using the BioMart (http://www.biomart.org/) data management system, we retrieved the gene content in the CNVRs identified. Out of the 25322 porcine Ensembl genes, 147 (Table S2 in File S1) were completely or partially overlapped with CNVRs, including 127 protein-coding genes, 11 pseudo genes, 3 miRNA genes, 3 snRNA genes, and 3 genes of other types. These genes are distributed in 38 (60.32%) CNVRs identified, while the other CNVRs do not contain any annotated genes.

To provide insight into the functional enrichment of the CNVs, Gene Ontology (GO) and Kyoto Encyclopedia of Genes and Genomes (KEGG) pathway analyses were performed with the DAVID bioinformatics resources v6.7 (Table S3 and Table S4 in File S1). The GO analyses revealed 29 GO terms, of which 20 were statistically significant after Benjamini correction. And the significant GO terms were mainly involved in olfactory receptor activity, sensory perception of smell or chemical stimulus, G-protein coupled receptor protein signaling pathway, cell surface receptor linked signal transduction, and other basic metabolic processes. In the KEGG pathway analyses, only one pathway, *i.e.*, olfactory transduction, was identified, which was statistically significant after Benjamini correction.

We compared the CNVRs identified in this study with the reported QTL regions and human disease gene orthologs in OMIM. Out of the 8315 QTL collected in the pig QTL database, 1364 (16.40%) were overlapped with the 61 CNVRs (96.83%) identified in this study (Table S5 in File S1). Since the total length of the CNVRs covers only 0.36% of the whole swine genome, there is a much greater QTL density coinciding with the CNVRs than we see in the genome as a whole. These CNVRs affecting a wide range of traits, such as growth, meat quality, reproduction, immune capacity and disease resistance. In addition, 6 human orthologous genes were also observed in 5 CNVRs (Table S6 in File S1). These genes associated with several human diseases, such as nicotine dependence, schizoaffective disorder, and alpha-1-antichymotrypsin deficiency.

### CNV validation by qPCR

In order to confirm the accuracy of CNV prediction, quantitative real time PCR (qPCR) was used to validate 12 CNVRs chosen from the 63 CNVRs detected in the study. These 12 CNVRs represent different predicted status of copy numbers (*i.e.*, loss, gain and both) and different CNVR frequencies (varied from 7.14 to 28.57%). Two pairs of primers (Table S7 in File S1) were designed for each CNVR and a total of 24 qPCR assays were performed. Out of the 14 samples genotyped, 13 were used in the qPCR assays. The positive predictive rate and negative predictive rates of the 8 CNVRs were calculated.

Out of the 24 qPCR assays, 13 (54.17%) were in agreement with prediction by PennCNV. When counting the CNVRs, 8 (66.6%) out of the 12 CNVRs (Table 2) had positive qPCR confirmations by at least one PCR assay. For the samples predicted as positive of the 8 CNVR, the proportions of confirmed samples (*i.e.* positive predictive rate) varied from 50% to 100%, with an average of 92.31%, whereas for the samples predicted as negative, the proportions of confirmed samples (*i.e.* negative predictive rate) varied from 0 to 100%, with an average of 33.33%.

**Table 2:** Results of quantitative real-time PCR analysis of the 8 confirmed CNVRs.

CNVR _ID	Chr.	Strart^a^	End^a^	Freq.	Type	Primer	Predicted positive samples			Predicted negative samples			Validated
							Sample	Sample	Positive	Sample	Sample	negative	
							detected	confirmed	predictive rate	detected	confirmed	predictive rate	
**7**	1	296533542	296809518	0.1429	gain	E1-2	2	2	1	11	9	0.8182	Y
						E2-1	2	2	1	11	11	1	Y
**50**	7	50327355	50750507	0.0714	loss	D1-1	1	1	1	12	0	0	Y
						D2-1	1	1	1	12	0	0	Y
**53**	7	122956303	123104663	0.1429	gain	H1-1	2	1	0.5	11	2	0.1818	Y
						H2-2	2	2	1	11	2	0.1818	Y
**11**	11	62514730	62841029	0.1429	loss	F1-2	2	2	1	11	0	0	Y
						F1-3	2	2	1	11	0	0	Y
**12**	11	70508069	71089190	0	gain	J1-3	–	–	–	11	7		N
						J2-1	2	2	1	11	4	0.3636	y
**13**	11	84740020	84849706	0.0714	loss	M1-2	1	1	1	12	4	0.3333	Y
						M2-1	1	1	1	12	4	0.3333	Y
**20**	14	3815879	3862162	0.1429	loss	B1-1	2	0	–	–	11	0	N
						B2-1	2	1	0.5	11	5	0.4545	Y
**27**	15	13218467	13325812	0.2857	loss	L1-1	–	–	–	9	–		N
						L1-3	4	4	1	9	6	0.6667	Y
**Average**									0.9231			0.3333	

a The Sus scrofa assembly (10.2) (http://www.ensembl.org/Sus_scrofa/Info/Index) was used to indicate the position of the CNVRs.

## Discussion

In the present study, using the PorcineSNP60 Beadchip, we identified 63 CNVRs (96 CNVs) in 14 individual of diverse populations, including six types of Chinese indigenous breeds, one Asian wild boar population, as well as three modern commercial breeds. As shown in Table 1, the CNV number detected per individual varies greatly, ranging from 2 (MS8) to 13 (D2). Chinese indigenous breeds have larger genetic diversity than European breeds [30], and it is expected more CNVs would existed in them. Contrary to expectations, in our study, fewer CNVs per pig were detected in Chinese indigenous and wild pigs (average 5.5) than in the modern commercial breeds (average 10.5). Similar result was obtained in the study of Chen et al. [24], in which highest CNVs per pig were found in Duroc. SNP probes in PorcineSNP60 BeadChip were mainly derived from sequence of four modern commercial pig breeds (Duroc, Landrace, Large White, Pietrain) [37], and SNPs among some paralogous genes of Chinese indigenous pigs could not be genotyped. It may be one reason leading to the difference of CNVs detected between Chinese indigenous breeds and modern commercial breeds.

We assessed our results by comparing with CNVRs previously reported [21], [22], [23], [24], [25], [26]. Of the total 63 CNVRs, 34 (53.98%) were completely or partially overlapped with CNVR detected by other studies (Table 3 and Table S8 in File S1), demonstrating about half of CNVRs identified in the study can be confirmed by other studies. As shown in the Table 3, the CNVR number overlapped with the 6 previous studies varies greatly, and 4 of them have only small number (1∼5) CNVRs overlapped with our study. The issue of low overlapping rates between different reports was also encountered in other CNV studies [28], [38], [39]. The potential reasons for the inconsistence between results of different studies lie in many aspects, such as the differences of samples in size and genetic background, different detection platforms and algorithms for CNV calling, and CNV (CNVR) definition between these studies as well as potential technical and random errors. In the present study, 14 samples of diverse breeds with broader genetic background were used, and it is expected that some new CNVRs would be detected in them. Due to the same detection platform and similar sample populations, the highest percentages of both overlapped CNVs count (49.21%) and sequence length covered (45.13%) were obtained with study performed by Chen et al. [24], which were performed using Porcine SNP60 array based on the 18 pig populations, including several Chinese indigenous breeds.

**Table 3:** Comparison between CNVRs detected in the study with those in the previous reports.

Study	CNVR detected in the previous studies							Overlaps with this study			
	Methods	Sample	CNVR	Range	Median	Mean	Total	Count	Percentage	Total length	Percentage
				(kb)	(kb)	(kb)	(Mb)		of count	(kb)	of length
**Fadista et al., 2008**	aCGH (385k)	12	37	1.74–61.92	6.89	9.32	0.43	1	1.59	0.41	0.00
**Ramayo-Caldas et al., 2010**	SNP chip (60k)	55	49	44.65–10715.82	170.96	754.59	36.97	2	3.17	215.61	2.16
**Wang et al., 2012**	SNP chip (60k)	474	382	5.03–2702.75	142.90	250.69	95.76	5	7.94	388.09	3.89
**Li et al., 2012**	aCGH (720k)	12	259	2.30–1550	98.74	65.07	16.85	2	3.17	718.74	7.20
**Chen et al., 2012**	SNP chip (60k)	1693	565	50.39–8102.06	252.71	247.55	139.87	31	49.21	4502.75	45.13
**Rubin et al., 2012**	Genome sequencing	117	1928	0.12–175.50	3.00	5.23	10.08	10	15.87	561.14	5.62
**This study**	SNP chip (60k)	14	63	3.20 –827.21	97.85	158.37	9.98	–	–	–	–

Note: The comparison was based on Sus scrofa assembly (10.2). For CNVRs based on the other porcine assembly, we firstly converted the data to current genome coordinates using the UCSC LiftOver tool (http://genome.ucsc.edu/cgi-bin/hgLiftOver).

In order to confirm these potential CNVRs, we performed qPCR for 12 randomly selected CNVRs from the total CNVRs identified in the study and 8 of them (66.67%) were validated successfully. The confirm rate was similar with previous studies [23], [25], [28], but a little lower than that reported by Ramayo-Caldas et al. [25] and Chen et al [24]. For the 8 CNVRs confirmed in the qPCR analysis, the average proportion of the positive predictive rate was 92.31%, demonstrating that, for the predicted positive samples, qPCR assays agree well with the PennCNV prediction. As for the discrepancy between qPCR assay and prediction, the most important reason is primers out of the actual boundaries of CNVs for some individuals. Due to the low marker density, non-uniform distribution of SNPs along pig chromosomes [37], CNVs called based on PorcineSNP60 BeadChip are usually large and with inaccurate boundaries. Beside the primers location used to validate the CNVRs, potential SNPs and small indels could also influence the hybridization of the qPCR primers in some animals, and result in unstable quantification values or reducing primer efficiency. Contrary to predicted positive samples, high negative predictive rate (average 33.33%) indicated that some predicted negative samples did not agree with the PennCNV prediction. In particular, for the CNVR#7, the negative predictive rate is as high as 81.82% and 100%. False negative is common in CNV detection, and has also been reported previously in pig and other mammalian species [23], [25], [27]. It can be explained by the stringent criteria of CNV calling which minimizes the false-positive, on the other hand leads to high false-negative rate.

Three out of the 8 successfully validated CNVRs contain functionally important genes. Two qPCR assays with primers located in two olfactory receptor 1J4-like genes (*LOC100623462* and *LOC100157267*) were used for *CNVR22* validation. The olfactory receptor gene superfamily is the largest in vertebrate genomes, which function in the reception of innumerable odour molecules in the environment. Previous studies have showed that CNVs are highly prevalent among human and other vertebrate *OR* genes [40], [41], [42]. Two qPCR assays with primers located in two defensins genes (*BD114* and *DEFB110*) were used for CNVR#50 validation. Defensins are one of the largest and most studied families of antimicrobial peptides. In addition to their antimicrobial activity, they are also thought to play fundamental roles in both innate and adaptive immunity in higher organisms. A qPCR assay with primers located in the *SERPINA3* gene was used for validation of CNVR#53. *SERPINA3* also known as a1-antichymotrypsin, is a typical acute-phase protein secreted into the circulation during acute and chronic inflammation. Variations in this protein's sequence have been implicated in Alzheimer's disease, and deficiency of this protein has been associated with liver disease [43], [44]. By impacting the gene product amount or the regulation of these genes, copy number change of these immune-related genes, such as defensins and *SERPINA3*, may have a great influence on pathogen monitoring and disease resistance of diverse pigs.

Most of the CNVRs identified in the study span QTL regions, which influence a wide range of traits, especially growth, meat quality, reproduction, immune capacity and disease resistance, indicating these CNVRs may play an important role in pig economically important traits and disease susceptibility. These CNVRs cover or overlap with a total of 147 genes. Functional analyses, such as GO and pathway, showed that these genes were mainly enriched in sensory perception of the environment, response to external stimuli and immunity, which is consistent with other previous studies [23], [27], [28], [45]. Additionally, several CNVRs spanned genes associated with several important human diseases. These demonstrated that the CNVRs identified in the study may play an important role in exploring the genetic basis of phenotypic variability and disease susceptibility among pigs.

In summary, we have performed a genome-wide CNV detection based on the PorcineSNP60 genotyping data of 14 pigs of diverse breeds. A total of 63 CNVRs were identified, which is an important complementary to the CNV map in the pig genome. Validation of 12 CNVRs of these CNVRs produced a similar confirm rate (66.67%) as previous CNV studies based on SNP arrays. Functional annotation revealed the CNVR identified have important molecular function, and may play an important role in phenotypic variation and are often related with disease susceptibility. However, only large CNVRs (Average length 158.37 kb) were identified using this SNP panel. As statistics of the size distribution of human CNVs in Database of Genomic Variants (http://dgvbeta.tcag.ca/dgv/app/home?ref=NCBI36/hg18), the CNVRs are most abundant in the 1 to 10 kb range, and the CNVR number decrease gradually when the the CNVR length larger or smaller than the range. Thus, the number of CNVs identified in this study is likely to be a greatly underestimation of the true number of CNVs in these pig genomes. Follow-up studies, using improved SNP arrays as well as other technologies, such as aCGH and next-generation sequencing, should be carried out to attain high-resolution CNV map.

## Supporting Information

File S1 **File includes Tables S1–S8.** Table S1: The detailed information of each CNVR identified in this study. Table S2: Annotation of genes in CNVRs detected in this study. Table S3: Gene Ontology of genes in CNVRs identified. Table S4: Pathway of genes in CNVRs identified. Table S5: QTLs harbored within or partially overlapped with identified CNVRs across the pig genome. Table S6: The CNVRs completely or partially overlapped genes in Online Mendelian Inheritance in Man annotations. Table S7: Information of the validated CNVRs and the primers used in quantitative PCR analyses. Table S8: Detail information between CNVRs detected in the study with those in the previous reports.
(XLS)
